# Low frequency Raman Spectroscopy for micron-scale and *in vivo* characterization of elemental sulfur in microbial samples

**DOI:** 10.1038/s41598-019-44353-6

**Published:** 2019-05-28

**Authors:** Christine Nims, Brandi Cron, Maxwell Wetherington, Jennifer Macalady, Julie Cosmidis

**Affiliations:** 1 Department of Geosciences, Pennsylvania State University, University Park, Pennsylvania, 16802 USA; 2 Materials Science Characterization Laboratory, Pennsylvania State University, University Park, Pennsylvania, 16802 USA

**Keywords:** Characterization and analytical techniques, Element cycles, Element cycles

## Abstract

Elemental sulfur (S(0)) is an important intermediate of the sulfur cycle and is generated by chemical and biological sulfide oxidation. Raman spectromicroscopy can be applied to environmental samples for the detection of S(0), as a practical non-destructive micron-scale method for use on wet material and living cells. Technical advances in filter materials enable the acquisition of ultra-low frequency (ULF) Raman measurements in the 10–100 cm^−1^ range using a single-stage spectrometer. Here we demonstrate the potency of ULF Raman spectromicroscopy to harness the external vibrational modes of previously unrecognized S(0) structures present in environmental samples. We investigate the chemical and structural nature of intracellular S(0) granules stored within environmental mats of sulfur-oxidizing γ-Proteobacteria (*Thiothrix)*. *In vivo* intracellular ULF scans indicate the presence of amorphous cyclooctasulfur (S_8_), clarifying enduring uncertainties regarding the content of microbial sulfur storage globules. Raman scattering of extracellular sulfur clusters in *Thiothrix* mats furthermore reveals an unexpected abundance of metastable β-S_8_ and γ-S_8_, in addition to the stable α-S_8_ allotrope. We propose ULF Raman spectroscopy as a powerful method for the micron-scale determination of S(0) structure in natural and laboratory systems, with a promising potential to shine new light on environmental microbial and chemical sulfur cycling mechanisms.

## Introduction

Elemental sulfur S(0) is prevalent across a diverse number of sulfide-rich environments from geothermal^1^ and cold sulfidic springs^2^, deep-sea hydrothermal vents^3^, to salt marshes^4^, marine sediments^5,6^, pyroclastic ash emissions^7^, mangrove swamps^8^, deep-sea hydrocarbon seeps^9^, and waste-water treatment plants^10^. Its presence in the environment is often associated with microbial oxidation of reduced sulfur compounds^11^. Elemental sulfur can also be formed through abiogenic processes when sulfide is oxidized by molecular oxygen, possibly catalyzed by oxidized metals^12^. S(0) plays a central role in the sulfur biogeochemical cycle, and is used as an energy source by a variety of sulfur-oxidizing, sulfate-reducing, and sulfur-disproportionating bacteria^13^.

Under Earth’s surface conditions, S(0) exists in many forms, from cyclic octamers (S_8_ rings) that crystallize in different structures or allotropes, to sulfur chains with varying numbers of S-S bonds, and polysulfides (S_n_^2−^)^5,14,15^. Characterization of S(0) in natural samples can be undercut by instabilities in its frangible chemical and structural properties. The allotropic enantiotropy of S_8_, its ability to catenate^16^, and many possible reduction or oxidation reactions make sulfur a difficult material for analysis. These difficulties have so far prevented a consensus on the speciation and structure of biological S(0) stored as intracellular globules in bacteria, which has been described as “S_8_ rings”^17^, “solid S_8_”^18^, “microcrystalline S_8_”^19^, “liquid sulfur”^20^, “a mixture of polysulfides and cyclooctasulfur”^21^, and sulfur chains associated with “unidentified organic residues”^22^. Considerations on the changing nature of microbial sulfur storage, where “the speciation of stored sulfur varies under different ecophysiological conditions”^21^, and the variance in sulfur speciation– cyclooctasulfur, sulfur chains, and polythionates – contingent on the sulfur-oxidizing metabolism^23^, suggest the necessity of *in vivo* methods to finally elucidate this question.

A series of publications has debated the application of synchrotron-based X-ray absorption near edge structure spectroscopy (XANES) at the sulfur K-edge to investigate the nature of intracellular sulfur globules^17,18,22–24^. Shortcomings of this method include possible distortions of the XANES spectra due to experimental artifacts^17^, the inability to discriminate different crystal structures of S_8_, and the infeasibility of *in vivo* measurements. X-ray diffraction (XRD) allows differentiation between S_8_ structures, but as a bulk method, it requires an abundance of material and does not provide spatial information on S(0) distribution within a sample. Micro- and nano-scale analyses by scanning electron microscopy (SEM), and transmission electron microscopy (TEM) also present significant drawbacks, since the vacuum conditions of the SEM and TEM chambers can cause the sublimation of S(0), while the electron beam can rapidly transform or burn sulfur material^19^.

Raman spectromicroscopy provides a non-destructive analytical technique in studies probing the chemical composition and speciation of biological sulfur. Raman scattering circumvents many of the problems associated with other characterization methods, as measurements can be collected on solid, liquid, and live samples at room temperature and atmospheric pressure. Additionally, Raman mapping produces high spatial (~1 μm) and spectral resolution analyses for detailed insights on the distribution and speciation of sulfur in the sample material. Characteristic internal vibrational (molecular) spectra make S(0) particularly easy to detect and characterize with Raman scattering^9,19,21,25–27^.

Innovation in Raman filter technology over the past decade has enabled newfound access to the ultra low-frequency (ULF) modes of the Raman spectrum^28^. The introduction of volume Bragg gratings has sanctioned measurements vicinal to the frequency of the laser line^29^, reaching approximately 10 cm^−1^. However, these new tools and advancements in the spectroscopy field have hitherto not been integrated within the earth and environmental science disciplines.

Most, if not all, biochemical, geomicrobiological, and kinetics studies using Raman analysis^8,19,21,27,30,31^ only focus on the properties of the S_8_ internal frequency regions of the Raman spectrum (>80 cm^−1^). In the absence of ULF spectral region, past research characterizing S(0) inclusions of sulfur bacteria through Raman spectroscopy relied on shifts in peak position, full width at half maximum (FWHM), and relative peak intensity^19,21^, which produced ambiguous conclusions on the chemical speciation of the biomineralized S(0), and no information on crystal structure. We argue that an examination of Raman spectra of sulfur beyond the intramolecular vibrational modes is required to discriminate between different possible sulfur forms in biological S(0).

Our work uses low-frequency (<100 cm^−1^) ULF Raman scattering as a tool to collect micro-scale spectral information on the external molecular structure and crystal phonons of S_8_. Combined with analyses on the internal mode peak assignments, this study explores novel spectral identification of metastable allotropic forms of S_8_, while investigating the *in vivo* speciation and structure of intracellular S(0) in a natural community of sulfur bacteria. Comprehensive inspection of Raman low frequency phases improves our ability to pinpoint and characterize the amorphous or crystalline forms of S(0) within environmental samples, while probing the potential sources of these sulfur particles.

Understanding the nature and distribution of sulfur could better constrain the conditions required for S(0) formation and preservation in biogeochemical processes and the mechanisms of microbial and chemical sulfur cycling.

## Results

### Reference spectra – External and internal S_8_ frequencies, molten sulfur, and polysulfides

#### Solid, S_8_ allotropes

**Figure 1:**
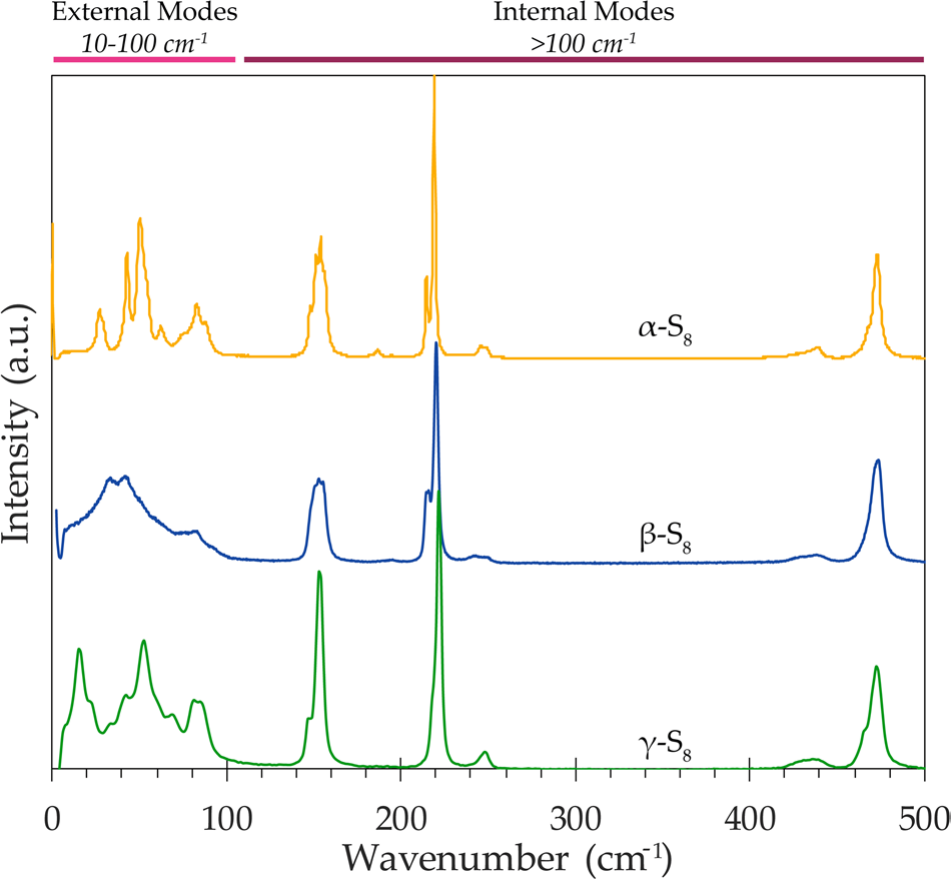
Raman spectra of reference standards of solid cyclooctasulfur allotropes: α-S_8_ (yellow), β-S_8_ (blue), and γ-S_8_ (green), all normalized to the 473 cm^−1^ peak. Wavenumber ranges for external and internal vibrational modes are indicated.

Raman spectroscopy detects the distinct vibrational modes of the S_8_ molecular structure. Figure 1 is a plot of the Raman spectra of three reference solid S_8_ allotropes. Low frequency solid S_8_ peak assignments measured in this study can be found in Supplementary Table 1.

All three solid S_8_ allotropes have similar internal, high frequency vibrations with a few minor spectral variations. Overall, scans of the reference S_8_ samples indicate characteristic Raman active peaks, with internal vibrations indicated by features at 153 cm^−1^, 220 cm^−1^, and 473 cm^−1^. The vibrational modes at ~150 cm^−1^ and 220 cm^−1^ represent asymmetric S-S bending and symmetric S-S bending respectively. The peak at 473 cm^−1^, with a shoulder feature at ~467 cm^−1^, represents S-S bond stretching of the S_8_ ring structure^32^. A broad feature at ~440 cm^−1^ is present in all solid allotropes which also corresponds to S-S stretching modes; the α-S_8_ spectrum presents a more defined peak at this position. The internal vibrations of metastable β-S_8_ mirror the other solid allotropes, however, the β-S_8_ asymmetric bending and the S-S stretching peaks present a minor broadening of FWHM in comparison to the other spectra. Additionally, in all normalized solid reference spectra, the relationship between the two bending features remains consistent, where the asymmetric bending peak (~150 cm^−1^) is invariably less intense than the symmetric bending mode (~220 cm^−1^). The three reference allotropes also present distinct asymmetric bending (~150) peaks, including variation in FWHM, relative intensities, and minor peak components, however, these peaks differences are inconsistent across several measurements

The intra-molecular vibrations of γ-S_8_ are consistent with the other S_8_ reference allotropes, with a minor but well-defined peak present at 248 cm^−1^ in both the γ-S_8_, and the α-S_8_, scans. γ-S_8_ presents a shoulder at ~216 cm^−1^ where α-S_8_ and β-S_8_ indicate a distinct peak in same position. The γ-S_8_ S-S stretching peak at 473 cm^−1^ is broadened and shows a more pronounced shoulder feature at 468 cm^−1^ than β- and α-S_8_.

The low frequency wavenumber region reveals major spectral differences between the solid S_8_ references. The external vibrations of α-S_8_ in the low frequency range are expressed by prominent peaks positioned at: 28 cm^−1^, 44 cm^−1^, 51 cm^−1^, 63 cm^−1^, and a doublet at 82 and 88 cm^−1^. Orthorhombic α-S_8_ is easily distinguished from the other S_8_ allotropes in the identification of a prominent librational feature at 28–30 cm^−1^ (depending on the scan)^33^. Several bands display doublet structures that can be ascribed to the crystal-field effects of rhombic S_8_^15^. The main low frequency vibrations of β-S_8_ yield broad, widened doublet feature with peaks at: 33 cm^−1^, 42 cm^−1^, 82 cm^−1^, and a shoulder at 60 cm^−1^ (Fig. 1). The low frequency spectrum measured in this work shares similar features with the β-S_8_ spectra published in previous Raman scattering studies^34,35.^ Here we present the first published γ-S_8_ Raman spectrum which includes both external and internal modes (Fig. 1). The inter-molecular vibrations of γ-S_8_ in the low frequency region include features at: 15 cm^−1^, 22 cm^−1^, 33 cm^−1^, 41 cm^−1^, 52 cm^−1^, 68 cm^−1^, and a doublet peak at 83 and 85 cm^−1^. The external diagnostic peak of γ-S_8_ at 15 cm^−1^ is the lowest wavenumber feature apparent in any of the solid S_8_ reference allotropes.

#### Molten S_8_ – Liquid sulfur

**Figure 2:**
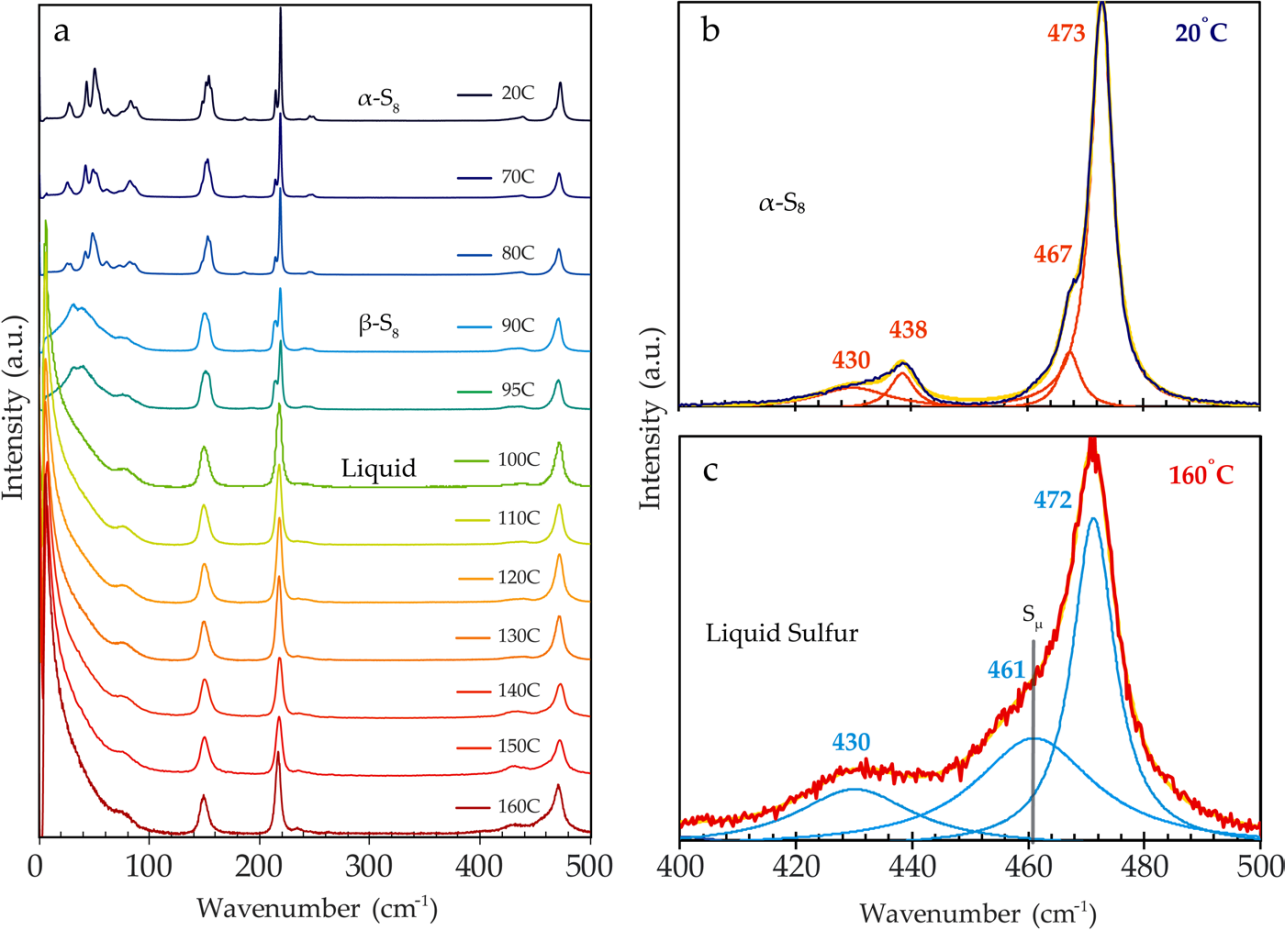
(**a**) Raman spectra from an *in-situ* experiment at increasing temperatures. The experiment begins with solid α-S_8_, with an observable transition to β-S_8_, and then a transition to liquid S_8_. Spectra are normalized to the 150 cm^−1^ peak; (**b**) A plot of the 20 °C α-S_8_ spectrum and **(c)** the 160 °C liquid sulfur spectrum normalized to the characteristic 473 cm^−1^ S_8_ feature, showing Lorentzian functions (orange and blue respectively) and the cumulative fit in yellow. Plot (**c**) shows the presence of a shoulder feature (461 cm^−1^), which denotes the accretion of disordered polymeric sulfur chains (S_μ_) in the high temperature liquid sulfur.

High temperature *in-situ* Raman experiments probing the composition and structure of molten sulfur are presented in Fig. 2. Measurements of powder α-S_8_ standard initiate the experiment, with Raman spectra acquired as temperature increases incrementally from 20 to 160 °C. The phase transition from α-S_8_ to β-S_8_ to liquid sulfur (Fig. 2a) produces extensive variation in the ULF region of the Raman spectrum, while it generates only subtle spectral changes in the internal frequencies. The loss of crystallinity and long-range order is apparent in the ULF spectra, as the definitive peaks of the α- and β-S_8_ lattice vibrations meld into a smooth, wing feature near the Rayleigh line in the molten sulfur scans. The wing feature is a spectral signal representative of amorphous, disordered materials, referred to as the Boson peak^36^, and it is a characteristic of liquid sulfur in the external modes. Supplementary Fig. 1 compares the spectrum of molten sulfur with the spectrum of the aluminum foil on which the sample was analyzed to verify sulfur as the source of the Boson peak signal. Additionally, the torsional vibration band at the crystal lattice boundary, which is a strong doublet peak in the α-S_8_ spectra at 82 and 88 cm^−1^, and a distinct peak feature respectively in the β-S_8_ spectra, morphs into a soft shoulder on the Boson peak in the molten sulfur spectra (Fig. 2a), indicating the loss of torsional vibration.

Subtle spectral changes occur in the internal vibrations as sulfur transforms from its solid to liquid state. Overall, a decrease in signal-to-noise ratio between the spectrum of crystalline solid (Fig. 2b) and the molten sulfur spectrum (Fig. 2c) can be attributed to the phase change and subsequent differences in the Raman scattering cross-section of the sulfur. The FWHM of the molten sulfur peaks increases marginally over the course of heating. The multiple Raman components of the 220 cm^−1^ S-S bending vibration disappear into a singular peak feature around 100 °C, the melting point of β-S_8_ (Fig. 2a). This peak remains relatively more intense than the S-S stretching peak at 473 cm^−1^ across all spectra in the *in-situ* experiment.

Several chemical physics, soft matter, and kinetics studies explore the unique liquid-liquid “λ” transition of sulfur at T_λ_ = 159 °C, where liquid sulfur polymerizes into long chain structures (S_μ_)^37–41^. This transition is illustrated by changes in the 473 cm^−1^ peak region at elevated temperatures, with the development of an expansive shoulder around 461 cm^−1^ in the 140–160 °C molten spectra (Fig. 2a,c). This feature has been associated with the polymerization of cyclooctasulfur rings, a process whereby the scission of S-S bonds and subsequent concatenation yield long polymeric diradical S_μ_ chains^37–40,42,43^. However, the higher frequency, 467 cm^−1^ shoulder of the the α-S_8_ standard in Fig. 2b, is assigned to an S-S stretching vibration of the α-S_8_ crystal in Raman polarization studies^33,44^. An increase in the intensity of the 430 cm^−1^ peak at high temperatures (>150 °C) (Fig. 2a,c) also signals the increasing polymer content and the progression of polymerization^45^.

Some polarization studies investigate the extent of sulfur polymerization by comparing the depolarization ratio of Raman lines in the high frequency range to identify differences in chain length and molecular weight species^38^. This study does not explore the depolarization ratio, but it notes the presence of symmetric stretch vibrations (461 cm^−1^) of the polymeric S_μ_ component present in the >140 °C molten spectra in Fig. 2c.

#### Polysulfides standard

**Figure 3:**
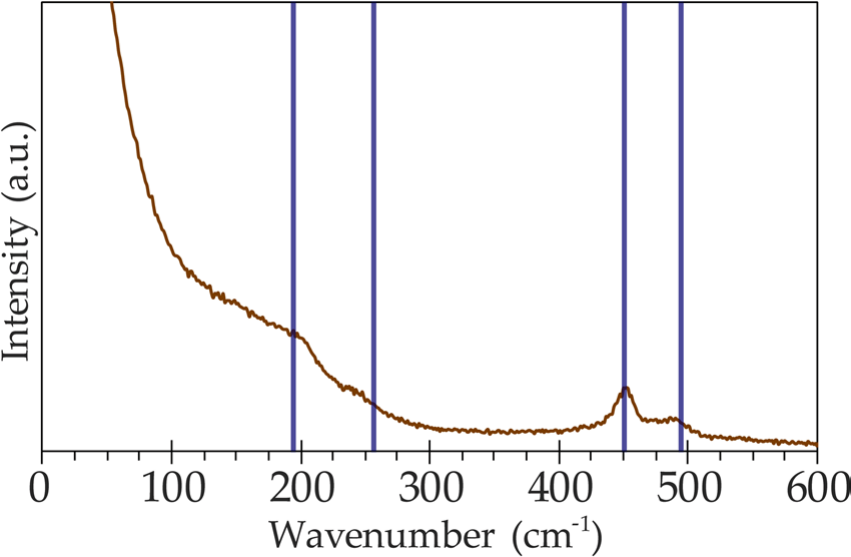
Spectrum of an aqueous polysulfide solution. Blue markers delineate positions of previously published polysulfide species bands^46,47^.

The Raman spectrum of a polysulfide solution is shown in Fig. 3. The plotted blue lines in Fig. 3 indicate published peak positions for Na_2_S_4_^46,47^ and similar values to the observed bands of S-S bond stretching in H_2_S_4_ polysulfanes^15^. The aqueous polysulfide spectrum presents generally weak and broad peaks, with the exception of the more pronounced 450 cm^−1^ feature, which is characteristic for aqueous Na_2_S_4_^46^. Our reference aqueous polysulfide scan lacks the diagnostic oxoanion shoulder at 337 cm^−1^, which suggests a pool of mostly reduced S_n_^2−^ species^47^. The undefined spectral wing in the low frequency range is also consistent with a liquid solution.

### S(0) storage globules in *Thiothrix* sp

#### Living *Thiothrix* cells

**Figure 4:**
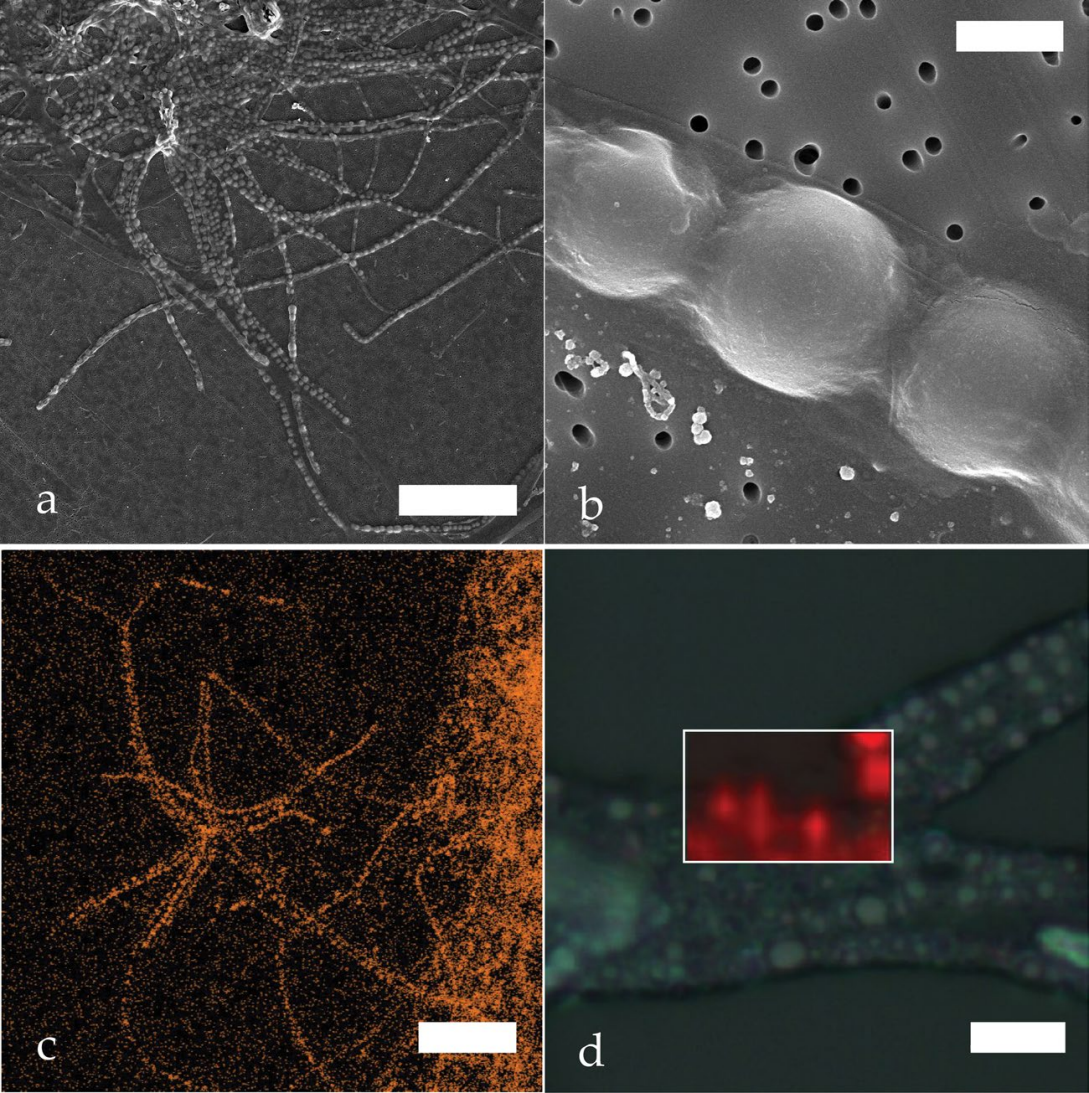
Intracellular sulfur globules in *Thiothrix* sp. (**a**) Scanning electron micrographs of *Thiothrix* filaments; (**b**) Close-up on *Thiothrix* intracellular sulfur globules; (**c**) SEM-EDS elemental map with *Thiothrix* sulfur globules highlighted in orange; (**d**) Raman map (white rectangle) of a bundle of *Thiothrix* filaments, where the areas mapped in red could be fitted with the biosulfur spectrum shown in Fig. 5. Scale bars: (**a**) 25 μm (**b**) 1 μm (**c**) 25 μm (**d**) 5 μm.

*In vivo* Raman spectroscopy of *Thiothrix* sp. was performed on samples examined immediately after field collection. Movement of the *Thiothrix* filaments under the microscope ensured the cells were alive throughout the *in vivo* Raman analysis. Complementary SEM analyses show that the sulfur globules, approximately 1 μm in diameter, stack within the enclosed sheath structure of the *Thiothrix* cells (Fig. 4). These sulfur inclusions rest within invaginations of the cytoplasmic membrane, as described in previous studies^48^.

**Figure 5:**
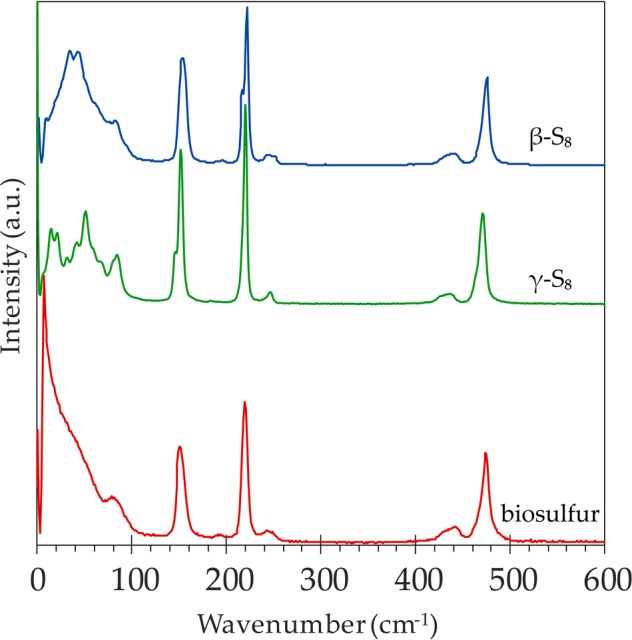
A plot of the sulfur allotrope spectra selected from mapped regions of several *Thiothrix* samples in varying conditions. The colors of the spectra correspond with the colors of the areas mapped in Figs 4, 6 and 7.

*In vivo Thiothrix* Raman spectra present the characteristic S_8_ structure previously described for the crystalline S_8_ standards and molten sulfur in the internal modes (>100 cm^−1^). The spectra present a definitive Rayleigh wing feature throughout the ULF region indicating amorphous or glassy disordered sulfur molecules (Fig. 5); the torsional peak around 80 cm^−1^ is positioned approximately five wavenumbers lower than the crystalline S_8_ allotropes (76 cm^−1^ instead of 82 cm^−1^) and it is relatively less pronounced in comparison to our reference S_8_ scans. The internal peaks have similar FWHM to the reference solid allotropic standards (Fig. 5). However, the individual S_8_ Raman components present in the main characteristic bending-stretching modes of solid allotropes, seen as shoulders on the 150 cm^−1^ and 220 cm^−1^ peaks, are absent in the biosulfur globules and could indicate differences in the intra-molecular bonds of the biosulfur structure.

#### Aged *Thiothrix*

To identify potential changes in the structure or speciation of the intracellular *Thiothrix* biosulfur with time after field collection, we measured samples stored in Falcon tubes in a refrigerator (at 5 °C), in original well water, for 17 days. Analyses of aged *Thiothrix* samples present spectral shifts compared with live samples, probably due to chemical changes occurring during storage of the samples. *Thiothrix* cells aged in native sulfidic well water at 5 °C likely experience oxidizing conditions through diffusion of oxygen from the headspace and the imperfect seal of the Falcon tube in which they are stored. For storage periods of hours to days, the sulfur inclusions are still visible within *Thiothrix* cells, and the spectral S_8_ bands are nearly indistinguishable from the spectra of fresh, living *Thiothrix*. However, after one or several months of prolonged storage, intracellular sulfur globules disappear, leaving behind empty sheaths. This could be due to the oxidation of S(0) by *Thiothrix* following the depletion of sulfide from the storage medium^49,50^.

**Figure 6:**
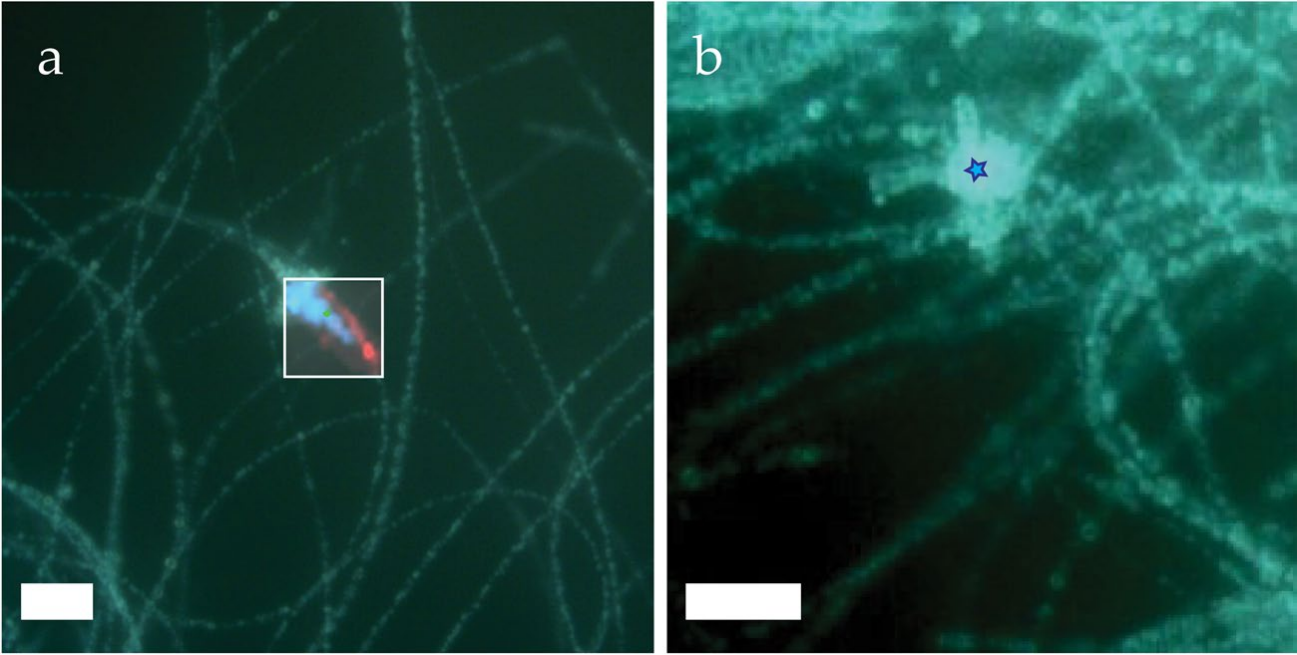
Aged *Thiothrix* in stored at 5 °C (**a**) Raman map (white rectangle) overlay on optical microscope image, showing extracellularly precipitated β-S_8_ (blue) adjacent to *Thiothrix* filaments containing globules of amorphous biosulfur (red); (**b**) Cluster of extracellular beta sulfur marked with blue star. Scale bars: (**a**) 10 μm (**b**) 5 μm.

Extracellular β-S_8_ crystals formed in a *Thiothrix* sample aged for 17 days in the original well water (Fig. 6). These sulfur precipitates, ranging in diameter from one to five micrometers, often develop proximal to the filaments still containing intracellular amorphous S(0) globules. The observations of S(0) oxidation and precipitation of extracellular S(0) with different structures argue for the necessity to limit time between sample collection and Raman analyses when characterizing sulfur in environmental microbial samples, as already discussed in another study^19^.

#### Dried *Thiothrix*

Raman analyses on freshly collected *Thiothrix* cells dried for two to three hours on a microscope slide indicate no detectable change in sulfur composition. The S(0) inclusions remain amorphous, stabilized within the cell membrane.

**Figure 7:**
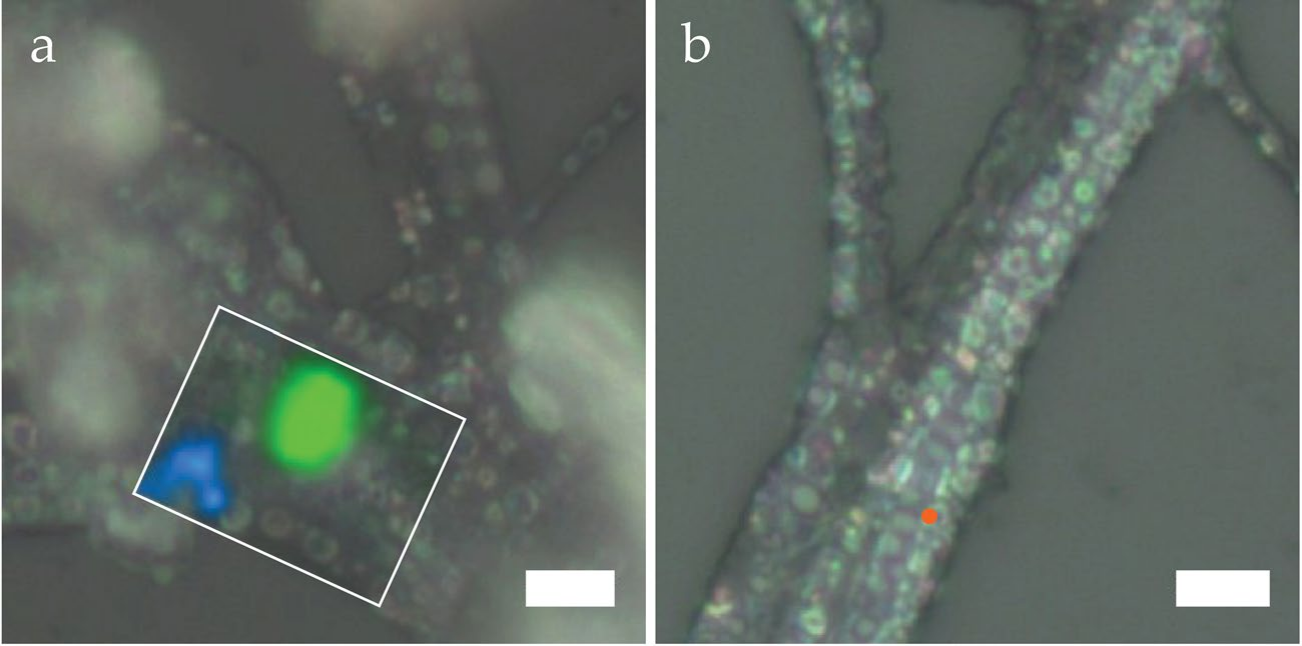
(**a**) Raman map (white rectangle) overlay on optical microscope image of externally precipitated β-S_8_ (blue) and γ-S_8_ (green) resting atop dried *Thiothrix* filaments (**b**) Two-week dehydrated *Thiothrix*, lodged with bright spherules mostly void of S(0). An intact globule (orange marker) with residual biosulfur could be identified. Scale bars: 4 μm.

Two-week dried *Thiothrix* samples, stored on microscope slides housed in foil-wrapped petri dishes, show extensive alteration in chemical and structural composition. Notably, Raman scattering on the bright intracellular features (Fig. 7b) produces featureless spectra from 0–1200 cm^−1^ with a strong fluorescence background signal, which could indicate some organic remainder (data not shown). These spherical inclusions are possibly empty vesicles of former sulfur storage globules^51^. Some filaments in the same samples however featured intact amorphous S(0) globules, albeit sparsely distributed, despite the weeks-long dehydration (Fig. 7b). This residual amorphous biosulfur in the dried samples co-occurs with >2 μm crystals of extracellular metastable S_8_ allotropes proximal to the cells. Figure 7a shows a dense cluster of γ-S_8_ adjacent to smaller β-S_8_ crystals resting atop S(0)-depleted *Thiothrix* filaments.

Raman analysis on the living, aged, and dried *Thiothrix* samples shows the absence of sulfur in any other oxidation state besides S(0). Sulfur oxoanion species, including sulfite, thiosulfate, and sulfate, have Raman band assignments in the 500 to 1100 cm^−1^ range, which were not observed in our scans performed into higher Raman frequency ranges. In particular, the symmetric S=O stretch vibration of sulfate at 960–1000 cm^−1^ ^52^ is undetectable in dozens of scans performed in this study, even in aged or dried samples.

**Figure 8:**
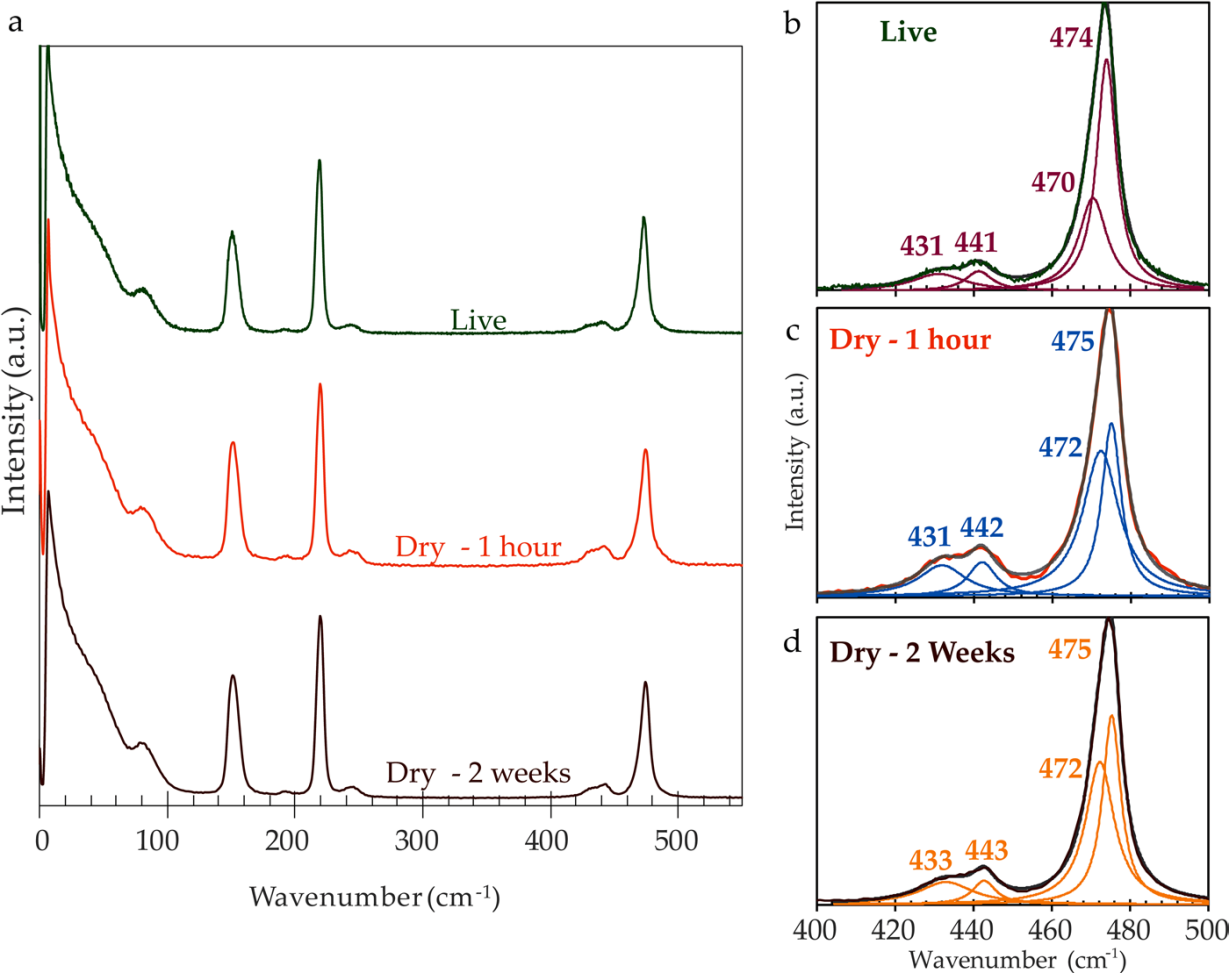
(**a**) The spectra of intact *Thiothrix* sulfur globules measured through a range of hydration states: live (top), dried on slide for one-hour (middle), and dried on slide for two weeks (bottom); (**b**–**d**) Peak fits in the 400–500 cm^−1^ range. Experimental data corresponds to color of the spectrum plotted in 8a; each fit profile is shown in gray; and Lorentzian peaks are shown in purple, blue, and orange. Spectra in (**a**) and (**b**–**d**) were normalized to the characteristic S_8_ 473 cm^−1^ peak.

A more detailed spectral exploration of intact sulfur globules remaining in the dried *Thiothrix* samples uncovers slight shifts in shoulder features within the low frequency modes and changes in the higher wavenumber internal vibrations (Fig. 8). The softened 80 cm^−1^ torsional peak of the living *Thiothrix* in Fig. 8a becomes slightly more prominent in the dried samples’ spectra. The relative intensities of the main S_8_ peaks are mostly conserved during drying, except for a slight increase in the intensity of the asymmetric S-S bending peak (150 cm^−1^) in the dried samples.

Figure 8b–d present peak fits in the 400–500 cm^−1^ wavenumber range of the spectra shown in Fig. 8a, and illustrate some changes in the S_8_ stretching modes of living versus dried (1 hour and 2 weeks) *Thiothrix* sulfur globules. The positions of the Lorentzian functions used to fit the spectra of the dried *Thiothrix* at 1 hour and 2 weeks inidicate vibrations of the S-S stretching peak, with components at 472 and 475 cm^−1^. These band positions are commonly attributed to the stretching of the S_8_ ring species^53^. These components are shifted into lower frequencies in the *in vivo Thiothrix* spectrum (470 and 474 cm^−1^) and this could indicate an increase in bond length. The spectral feature at 470 cm^−1^ shown in the *in vivo* sample spectrum can be attributed to either short sulfur chains, designated as S_μb_^39^, or to the S_8_ ring species^40^. The *in vivo* and dried *Thiothrix* spectra lack the diagnostic shoulder feature of long, polymeric sulfur chains (S_μ_) at 460 cm^−1^, prominent in the molten sulfur shown in Fig. 2b.

Peak fits of the *in vivo* and dried *Thiothrix* samples show two features at approximately 430 and 440 cm^−1^ in Fig. 8b–d. The positions of these stretching vibrations are consistent between the *in vivo* and 1 hour dried *Thiothrix*, but the peak positions shift to a slightly higher range (1 to 2 cm^−1^) in the 2 weeks dried sample. This trend could coincide with the drying process.

## Discussion

The present study highlights the advantages of using low-frequency Raman in the detection and characterization of elemental sulfur in microbial and environmental samples. This work supplements the limited literature on sulfur Raman external modes^33–35,44^, and presents the first low-frequency Raman spectrum of γ-S_8_ published so far. Several (geo)microbiological investigations have used Raman spectromicroscopy to confirm the presence of S(0) in environmental samples or laboratory cultures^8,9,19,21,26,27^. However, these previous studies did not include low-frequency measurements, limiting analyses of the phonon vibrations of the crystal lattice and hence structural determinations of the sulfur. Indeed, while only minor differences can be observed in the high frequency (>100 cm^−1^) Raman signature of different S_8_ allotropes, Raman measurements below 100 cm^−1^ enable clear determination of S_8_ crystalline structures, as well as non-crystalline forms of sulfur (Figs 1 and 2). While structural characterization of crystalline S(0) can also be achieved by bulk XRD analysis, this method is unsuitable for the detection of amorphous or liquid S(0), nano-cryalline S_8_ allotropes, and allotropes present in small quantities. For instance, our XRD analysis of aged *Thiothrix* samples failed to detect crystalline S_8_ phases, while our ULF Raman measurements revealed the presence of micron-sized β-S_8_ crystallites (Fig. 6). Furthermore, Raman spectromicroscopy, as a micron-scale method, enables spatial localization (mapping) of the analyzed particles and determination of their morphology, and can potentially be correlated with sub-micron scale imaging techniques such as SEM for high resolution analyses. Spatial correlation of particle morphology and S(0) crystal structure can also be achieved by electron diffraction methods, but only ULF Raman spectromicroscopy enables such investigations to be performed on wet or live samples under room temperature and atmospheric pressure conditions. The importance of *in vivo* and wet analyses when characterizing the structure of microbial or environmental S(0) was demonstrated here by the precipitation of extracellular monoclinic β-S_8_ and γ-S_8_ crystals in aged and dried *Thiothrix* samples (Figs 6 and 7).

Application of ULF Raman spectromicroscopy to S(0) detection and characterization in natural samples paves the way for future investigations of metastable monoclinic S_8_ allotropes in the environment. β- and γ-S_8_ have so far only been described in a few low-temperature natural settings^2,54,55^. It was recently demonstrated that β- and γ-S_8_ can be formed by sulfide oxidation in the presence of organics, through a process called S(0) organomineralization^56^. The stabilizing effect of organics on monoclinic S_8_ allotropes could explain the precipitation of β- and γ-S_8_ in the proximity of the microbial cells in our aged and dried *Thiothrix* samples (Figs 6 and 7). In the future, a more widespread application of low-frequency Raman analyses on natural samples might reveal that this process is more frequent than previously thought.

Further ULF Raman measurements can also be used to explore complex interactions or conditions promoting the precipitation of metastable S_8_ allotropes. Hydration sequences appear to impact the formation of γ-S_8_ in the environment. Localities experiencing cyclical hydration-dehydration phases host conditions favorable to γ-S_8_ crystallization: in evaporitic systems where gelled EPS matrix embeds crystals in cyanobacteria biofilms perched atop the water table^55^, in supraglacial perennial subsurface springs where EPS deposits encase sulfur rhombs^54^, in sewage sludge experiments where micro-crystals encrust dehydrated sewage granules^57^, in our experimental dried *Thiothrix* sample where γ-S_8_ particles form adjacent to cellular sheaths, and in other dried microbial microscope samples that yield prismatic, spindled sulfur crystals^19^. Low frequency investigations could help determine potential links between drying processes and the precipitation of metastable gamma sulfur in environmental samples.

Low-frequency Raman spectromicroscopy can also be employed to solve uncertainties in the ongoing debate on the composition of microbial biosulfur. This question has been the object of several studies using synchroton-based X-ray absorption spectroscopy (XANES) on a variety of sulfur bacteria, without resulting in a consensus. It has, for instance, been proposed that microbial sulfur was composed: of sulfur chains capped by unidentified organic residues^22^, simple solid S_8_^18,^ globules of varied speciation – cyclooctasulfur, sulfur chains, and polythionates – contingent on the sulfur-oxidizing metabolism^23^, and a core of S_8_ crowns enclosed in a hydrophilic envelope^17^. Analytical difficulties of XANES techniques leading to spectral distortions and artifacts further complicate this debate^17^. Our *in vivo* Raman analyses of intracellular S(0) globules in *Thiothrix* sp. showed diagnostic features of the internal vibrational modes of solid S_8_, in addition to a Boson peak in the external modes, indicative of disordered material. This suggests that globular biosulfur lacks long range order, countering previous Raman findings of microcrystalline biosulfur deposits^19,58^. The low frequency spectra of the *Thiothrix* biosulfur resemble the spectral features of liquid sulfur measured in our *in-situ* high temperature experiment (Fig. 2). However, the contribution of long polymeric sulfur, visible by the pronounced shoulder at 461 cm^−1^ in liquid sulfur – is absent from the biosulfur spectra. The 470 cm^−1^ peak observed in live *Thiothrix* cells could signal shorter polymeric chain species^38,39^, but this spectral component has also been assigned to S_8_ ring species in other work^40^. Considering the difficulty in resolving or assigning the 470 cm^−1^ peak, our analyses suggest that intracellular S(0) globules in *Thiothrix* sp. are mostly composed of disordered S_8_ rings, with the possible presence of short polymeric diradical species.

Another Raman study identified S_8_ as the main S(0) form in sulfur globules produced in *Beggiatoa* filaments, but only in subpopulations located at the sulfide-oxygen interface of gradient tubes or in early growth stage cultures^21^. *Beggiatoa* mats forming in a deeper sulfide-rich, anoxic zone, and freshwater gradient cultures presented S_8_ Raman active peaks with an additional feature at 272 cm^−1^, and a range of S-S stretching vibrations (~470 cm^−1^) respectively, suggesting a composite of S_8_ rings and linear polysulfides (S_n_^2−^) within the globules^21^. The characteristic polysulfide or polysulfane peaks were not detected in our Raman spectra of live *Thiothrix*, and these differences in polysulfide content could clue different S(0) metabolic pathways in *Beggiatoa* versus *Thiothrix*. Unlike *Thiothrix*, *Beggiatoa* can reduce stored S(0) into sulfide under short-term anoxia^59,60^, which could explain the presence and detection of polysulfide intermediates in the *Beggiatoa* measurements. That said, the transience and reactivity of polysulfide anions make them challenging to analyze, and inter-ion interactions in sulfide solutions can perturb the vibrational frequencies^47^ – arguing for a multidimensional approach in measuring S_n_^2−^ species.

XRD studies have suggested the presence of “liquid-like” sulfur in extracted microbial sulfur globules^20^, though these results face skepticism over the stability of liquid sulfur at room temperature and pressure^18^. Recent XANES data suggest that biosulfur particles have a density of 2.0 g.cm^−3^, overturning earlier assumptions of low, 1.2 g.cm^−3^ density globules and assigning a density value akin to crystalline α-S_8_^17^. The more recent assessment of biosulfur density – though contentious – supports our results suggesting globules dominated by closely stowed S_8_ crowns. Overall, our results are in agreement with other studies and Raman analysis indicative of intracellular microbial sulfur in the form of compact S_8_ rings^26,50,61^. Raman external mode vibration measurements in this work augment the current understanding of intracellular S(0) globules by verifying the disordered, amorphous inter-molecular structure of encapsulated S_8_ rings.

This proof-of-concept study demonstrates the capacity of low frequency Raman spectromicroscopy to reveal micro-scale S(0) speciation and structure in environmental and biological samples. Our work opens up the opportunity for an examination of the conditions and mechanism of preciptation and stabilization of metastable sulfur allotropes in the environment, and for future studies on the composition, formation and use of S(0) inclusions in different sulfur bacteria.

## Materials and Methods

### Sample collection and preparation

#### Microbial mats

Samples of microbial mats dominated by *Thiothrix* sp. – a sulfur-oxidizing, Gammaproteobacterial genus – were collected from an abandoned oil and gas well in Centre County, Pennsylvania. Fluorescence *in situ* hybridization (FISH) and cell morphology were used to identify the primary mat populations. FISH was carried out as described in Macalady *et al*. (2006)^62^ with commercially synthesized oligonucleotides (Sigma-Genosys). Slides were counterstained with 4′,6′-diamidino-2-phenylindole (DAPI). Microbial mat samples were dominated by rosettes of thin filaments, 1–1.5 μm in diameter, with intracellular S(0) inclusions that hybridized with the EUBMIX and GAM42a probes. FISH images are shown in Supplementary Fig. 2.

Several sampling excursions were performed from winter (December 2017) through spring (late May 2018). An estimated 60 mL of the microbial mat proximal to the sulfidic outflow was pipetted into sterile Falcon tubes during each site visit. Samples collected and analyzed on the same day were centrifuged at low speeds (<5000 rpm) and rinsed three times with ultrapure 18.2 MΩ water. Samples assessed in subsequent days were stored at 5 °C with oxygenated headspace in original well water. These samples were rinsed with ultrapure 18.2 MΩ water on the day of analysis. All samples were manipulated in a biosafety cabinet under laminar flow conditions to minimize contamination.

Preparation of living cells for *in vivo* Raman spectroscopy analyses mirrored the procedure used by Berg *et al*. (2014)^21^: a 20 μL droplet of rinsed sample was positioned on a glass microscope slide and secured by a taped coverslip to preserve the bacterial micro-habitat during data collection. “Dried” samples used un-sealed coverslips and were dry prior to analysis. We tested the repeatability of the Raman measurements on several collections of *Thiothrix* samples and on all sulfur standards.

### Raman spectroscopy

Raman spectroscopy of all samples and standards was conducted using a Horiba LabRam HR Evolution Raman Vis-NIR spectrometer coupled with a HeNe 633 nm laser source, an ideal excitation energy for applications aiming to minimize sample damage and requiring increased wavelength stability. The LabRam system utilizes an Olympus BXFM-ILHS optical microscope and IDS uEye 3 MPix video camera/software. BragGrate notch filters allowed the collection of low frequency (5–10 cm^−1^) signal. Most spectra in the study were collected using a groove density of 600 g/mm; 1800 g/mm grating was employed on certain samples and on all standards to better resolve both ULF vibrations and internal mode spectral features. The confocal pinhole aperture was set at 50 μm, providing a spectral resolution of ~0.8 cm^−1^. The acquisition range of the spectrometer was centered at 456 cm^−1^ to incorporate anti-Stokes and Stokes measurements (−200 cm^−1^ to 1000 cm^−1^) and verify features in the low frequency range. Reference spectra plotted in both regions can be found in Supplementary Fig. 3. The spectrometer was calibrated with a silicon standard prior to each Raman session.

The point measurements and Raman maps were carried out using either an apochromatic 50x or a 100x objective, while the reference standard spectra were collected using a 50x long working distance (LWD) objective. This analysis achieved a spatial resolution of approximately 1 μm. Point spectra were collected with two accumulations and 10 to 15-second exposure time; Raman mapping was conducted with one, 10-second accumulation, scanned in defined 0.75–0.80 μm x-y steps on an automated stage. Laser power was approximately 1–1.3 mW at the sample for both single point scans and mapping to avoid sample damage. Power levels documented in different sulfur-Raman studies provided a framework for the basic parameters in this research^19,21^. Additionally, to avoid potential photothermal effects and to address potential overlap with the absorption edge of long elemental sulfur chains, several power percentages were tested to optimize laser threshold power.

The LabSpec6 platform was used to process Raman spectra. Baseline correction was performed on raw spectra using polynomial fitting in LabSpec6 to remove fluorescence and background signal. Raman mapping incorporated the Classical Least Squares (CLS) fitting function in LabSpec6 to highlight the distribution of sulfur allotropes within a mapped region of interest. A representative spectrum was selected as the reference per each mapped sulfur component. Overlays of Raman maps on optical images provided visual correlation between the microscopic features and the molecular spectra. Optical microscope images were exported from LabSpec6 and processed using the ImageJ package.

The OriginPro software package enabled the nonlinear curve fitting of certain spectral features. Spectra were fit to Lorentzian peaks using a method based on the Levenberg-Marquardt iterative algorithm in order to detect overlapping features in the high frequency regions^63^.

### Reference sulfur compounds

#### *S*_8_*Allotropes*

All crystalline sulfur solids were recorded with Raman point measurements. The same samples were concurrently analyzed with X-ray powder diffraction to confirm their crystal structure (Supplementary Fig. 4).

Sulfur powder (Alfa Aesar 99% pure sulfur) provided the standard for orthorhombic, α-S_8_ sulfur. Orthorhombic sulfur is the thermodynamically stable allotrope at room temperature and pressure (Supplementary Fig. S5). For Raman spectroscopy, α-S_8_ powder was measured on a glass slide.

β-S_8_ is a metastable S_8_ allotrope that forms at temperatures above 96 °C (see Supplementary Fig. S5). β-S_8_ can be obtained from the quenched melt of molten α-S_8_^39^ and this monoclinic sulfur commonly forms elongated, pointed crystals. Approximately 10 grams of pure sulfur power (Alfa Aesar 99%) were emplaced in a test tube and melted with a torch. The liquified sulfur was quenched in ice water and formed a dark brown, tacky solid. Approximately one cm^2^ of this quenched sulfur was analyzed with Raman on a glass slide and by XRD within 30 minutes of solidifying to avoid re-crystallization of α-S_8_.

γ-S_8_ is a rare monoclinic allotrope of S_8_ that forms yellowish-white elongated, spiny crystals^64^. Akin to β-S_8_, gamma sulfur is stable at high temperatures and pressures (Supplementary Fig. S5). The slow oxygenation of a sulfide solution (500 μM Na_2_S, Spectrum Chemical) containing yeast extract (5 g.L^−1^, Fisher Scientific) for three weeks yielded stable particulate γ-S_8_ in a process described as S(0) organomineralization^56^. This precipitate was collected by centrifugation and rinsed with 18.2 MΩ water. A 20 μL volume of this concentrated slurry was pipetted onto a glass slide and allowed to dry before Raman investigation. XRD analyses confirmed the presence of γ-S_8_ as the only form of crystalline S(0) in two separate samples prepared through the same organomineralization protocol.

#### Molten sulfur

*In-situ* molten sulfur experiments were conducted on the same Horiba LabRam HR Evolution setup paired with a Linkam temperature-controlled stage (HFS600E) and the LNP95 cooling pump. Nitrogen gas was used to purge the stage system headspace. A 50x LWD objective was used with the Linkam stage. The stage was calibrated using a thermocouple to align actual temperature measurements with the recorded output of the Linkam control system. Synchronous temperature adjustments were recorded, corresponding with each incremental measurement of the heating experiment.

Sulfur powder (Alfa Aesar 99% sulfur) was loaded on a section of aluminum foil, enclosed, purged with N_2_, and then heated on the silver block stage body. The experiment was initiated at 70 °C, followed by 10 °C increases, to reach a final temperature of 160 °C. Additionally, the pivotal temperature point of allotropic conversion from α-S_8_ to β-S_8_ was measured at 95 °C^65^. Sample drying could not be mitigated with this stage configuration. Sample loss and the evaporation of molten sulfur above 170 °C prevented higher temperature measurements.

#### Polysulfides

A polysulfide reference solution was formulated following the recipe used in Berg *et al*., (2014), where a solution of 5.8 g of elemental sulfur per 100 mL 18.2 MΩ water was mixed with 5.06 g Na_2_S · 9H_2_O at a final pH of 9.5 in an anaerobic chamber and passed through a 0.2 μm filtration system^21^. Press-to-seal silicone isolators (Electron Microscopy Services) were used for Raman measurements. Duoscan averaging mode captured aqueous polysulfides spectra acquisitions over a 30 μm^2^ swath of sample, at approximately 1.3 mW, using a 200 μm confocal hole, 50x LWD objective, 633 nm laser, and 600 gr/mm.

### Complementary techniques

Scanning electron microscopy supplemented Raman data with high resolution imaging of the microbial samples. Secondary electron images were generated at an accelerating voltage of 7 kV, with a working distance (WD) of approximately 3 mm, using an FEI Nova NanoSEM630 instrument. Energy dispersive spectroscopy (EDS) analyses were performed at 15 kV and a 5 mm WD using an Oxford Instruments UltimMax detector and the data were analyzed using the Oxford AZtec platform. EDS maps and spectra confirmed microbial sulfur globules content and distribution.

For SEM/EDS analysis, samples were rinsed with 18.2 MΩ water, and deposited onto 0.22 μm Whatman Nucleopore filters, using a syringe filter holder dispensing system (25 mm, Pall Laboratory). Filters were placed on double-sided carbon tape atop aluminum stubs. All samples were sputter coated with iridium (12 nm) to prevent charging.

X-ray Diffraction data was collected on rinsed samples deposited on a single crystal miscut Si holder. Samples were analyzed using a PANalytical Empyrean diffractometer paired with a PIXcel^3D^ detector, and CuKα (λ = 1.5406 Å) incident X-ray radiation. Scans were conducted over a 2θ range of 5–70°, and analyses used a step size of 0.025°, a time of 96.4 seconds per step, and a current density of 40 mA. X-ray diffraction data were analyzed with Jade software. Recorded spectra were compared with reference spectra form the International Centre for Diffraction database.

## Supplementary information

Supplementary Information

## Data Availability

The datasets generated and/or analyzed during the current study are available from the corresponding author on reasonable request.
