# Supplementary material for: Low frequency Raman Spectroscopy for micron-scale and in vivo characterization of elemental sulfur in microbial samples
Source: Sci Rep. 2019 May 28;9:7971. doi: 10.1038/s41598-019-44353-6 (PMC6538736; doi:10.1038/s41598-019-44353-6)
Supplement: Supplementary file 1 — Supplementary Information [file 41598_2019_44353_MOESM1_ESM.pdf]

Supplementary Information

Low frequency Raman Spectroscopy for micron-scale and *in vivo* characterization of elemental sulfur in microbial samples

Christine Nims [1], Brandi Cron [1], Maxwell Wetherington [2], Jennifer Macalady [1], and Julie Cosmidis [1*]

[1] Department of Geological Sciences, Pennsylvania State University, University Park, Pennsylvania 16802, USA 1; jxc1158@psu.edu

[2] Materials Science Characterization Laboratory, Pennsylvania State University, University Park, Pennsylvania 16802, USA

* Correspondence: jxc1158@psu.edu; Tel.: + 814-865-7764

**Mode**	**α-S_8**	**β-S_8**	**γ-S_8**
Lattice – Libraations and Translations	28	33	15
	44	42	22
	51	60 (shldr)	33
	63		41
			52
			68
ν9	82, 88	82	83, 85

Table S1. A table of low frequency measurements of solid sulfur references (α-, β-, and γ-S8) recorded in this study. Data shown in cm^{-1}.

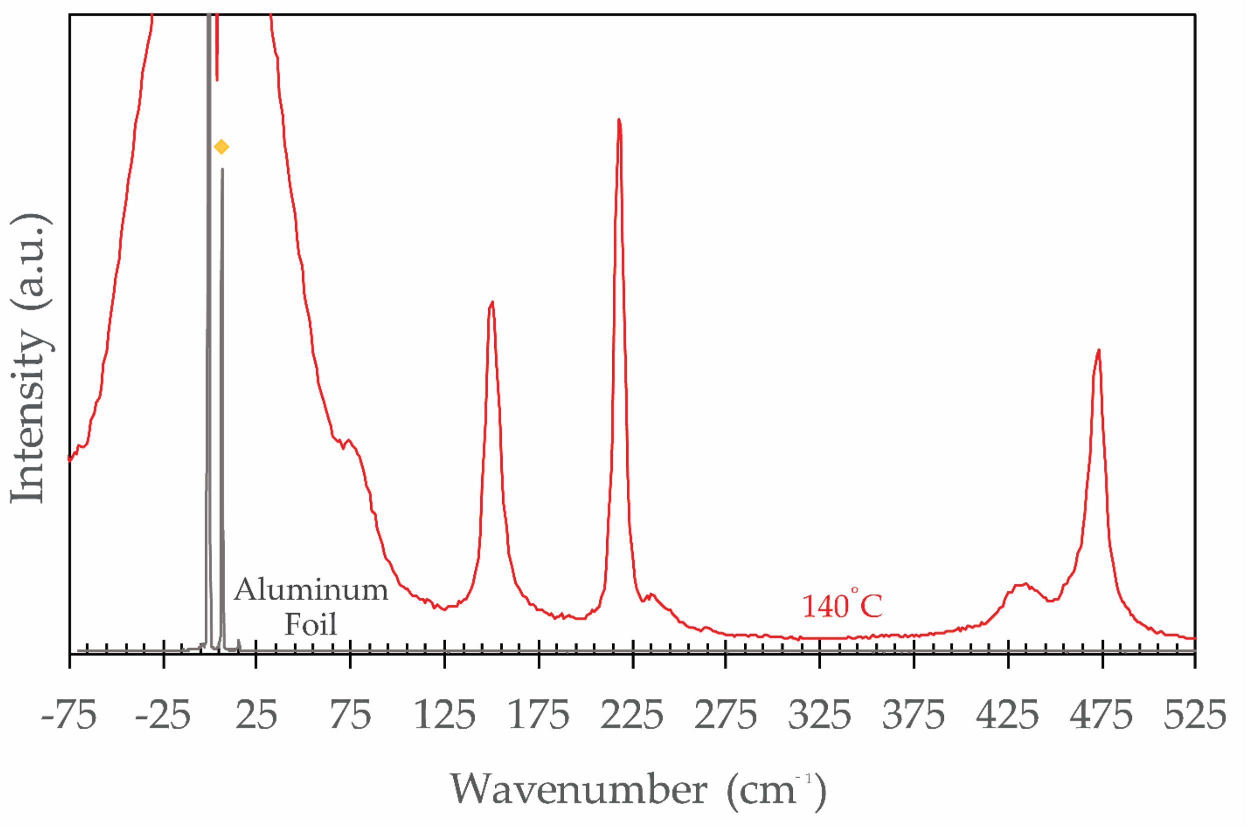

Figure S1. Molten sulfur (140˚C) spectrum in red plotted with the spectrum of aluminum foil in gray. The low frequency (0 – 100 cm^{-1}) Boson peak apparent in the liquid sulfur spectrum is not present in the aluminum foil spectrum, proving this spectral envelope is unrelated to the laser. The spurious, sharp peak (yellow diamond) in the foil spectrum at ~6 cm^{-1} is a plasma line from the He-Ne laser.

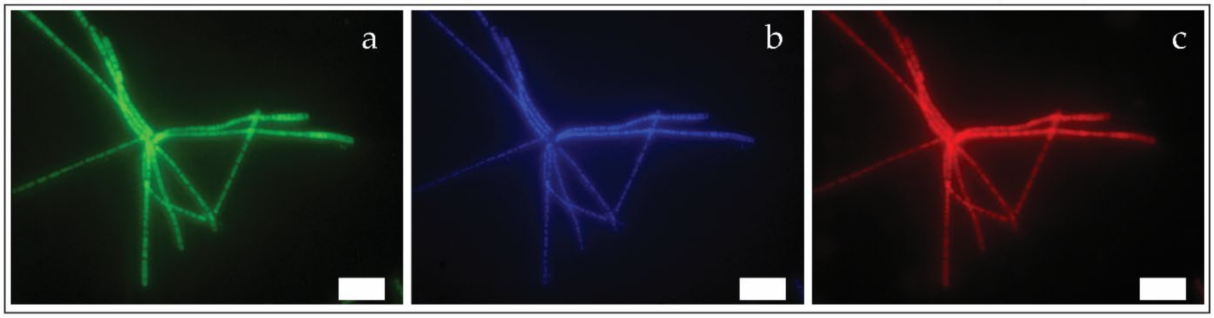

Figure S2. Fluorescence In Situ Hybridization (FISH) analysis of *Thiothrix* streamers used in Raman experiments. (**a**) EUBMIX (green) targeting all Bacteria, (**b**) DAPI (blue) targeting all DNA, and (**c**) GAM24a (red) targeting *Gammaproteobacteria* including *Thiothrix* spp.. Scale bar: 10 μm.

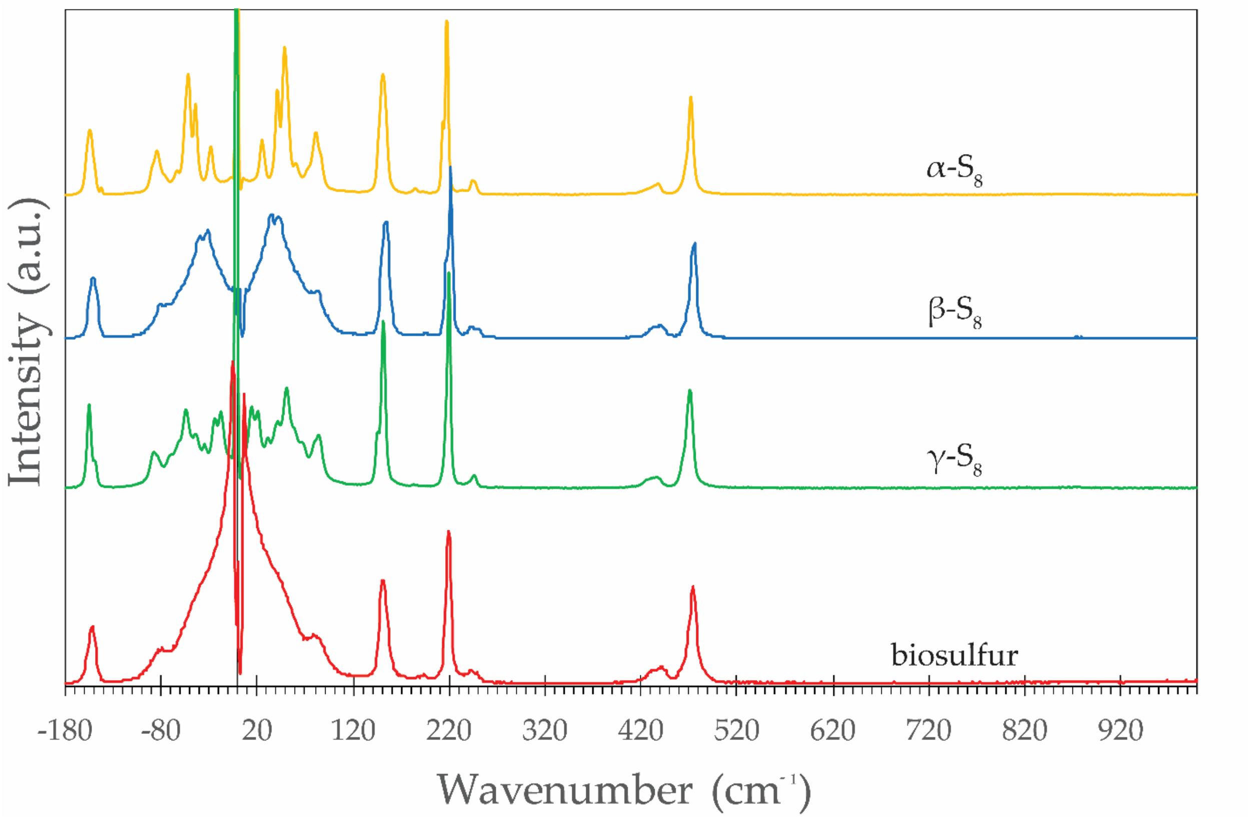

Figure S3. Solid S_8 standards (α-, β-, and γ-S8) and *Thiothrix sp*. spectra, including both Stokes and anti-Stokes measurements.

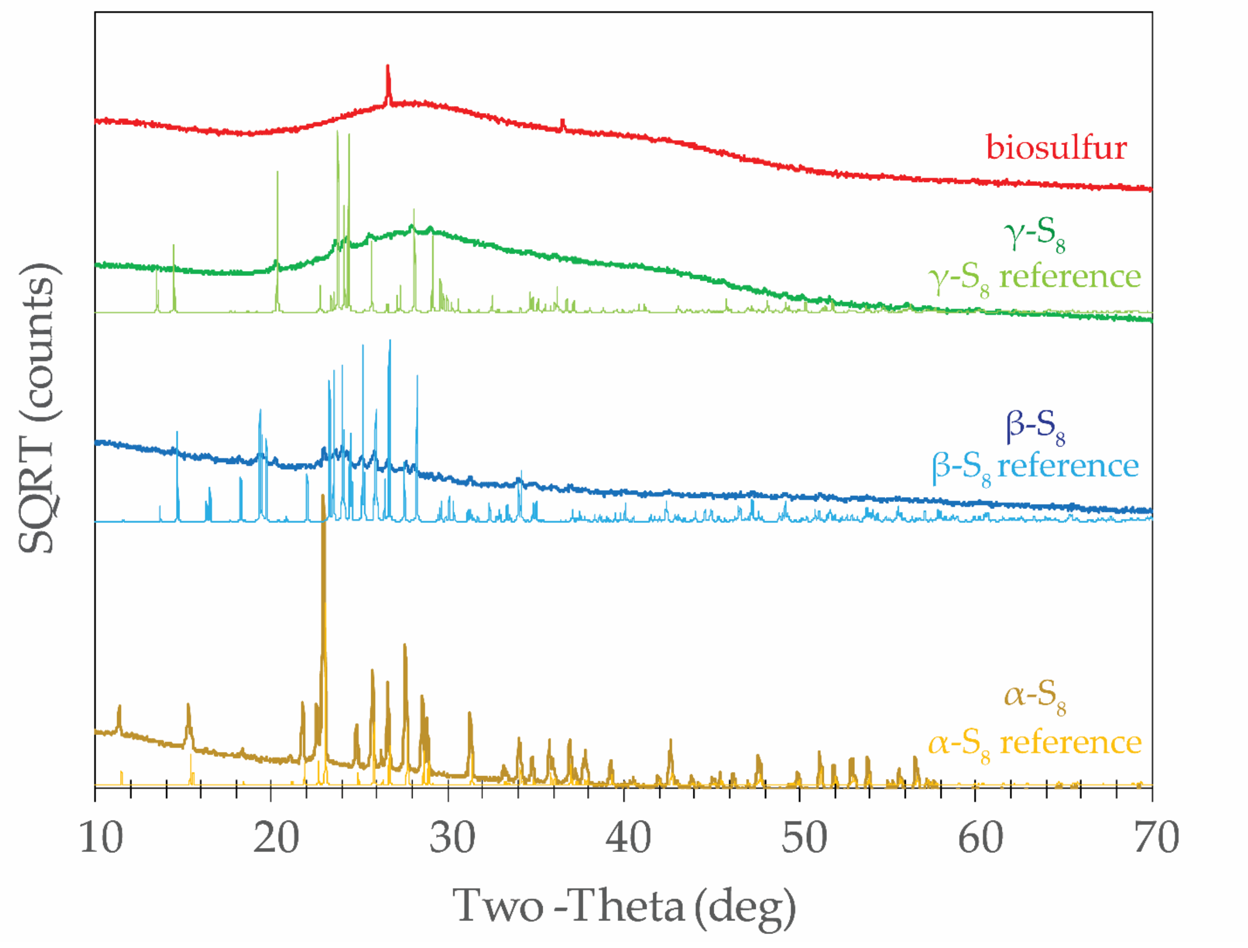

Figure S4. The X-ray diffraction patterns of *Thiothrix* sp. biosulfur and the solid S_8 Raman standards (α-, β-, and γ-S8), aligned with respective XRD reference patterns. The peaks present in the biosulfur spectrum are quartz/SiO_2, still intact in the environmental sample.

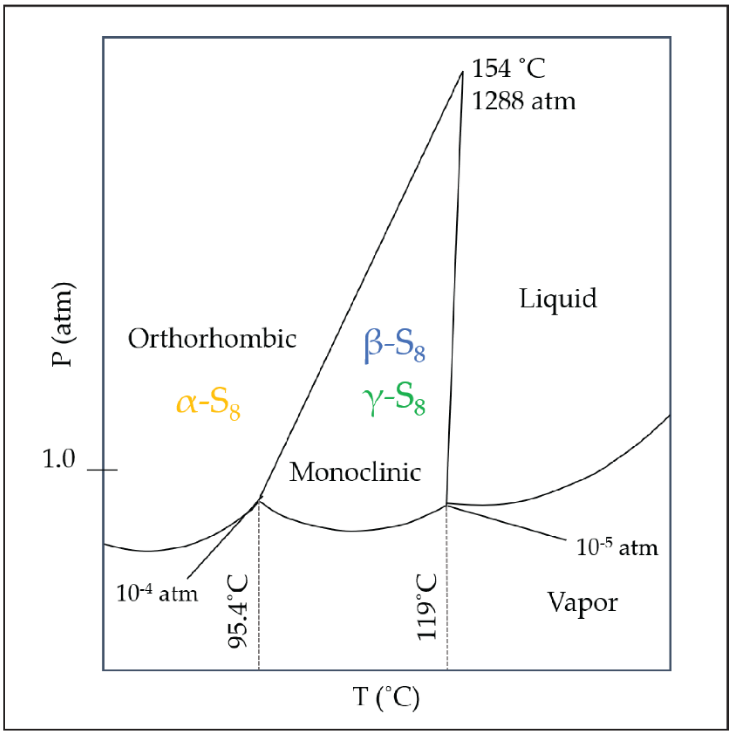

Figure S5. A sulfur temperature-pressure stability diagram, adapted from Figure 1 of Marriott et al. (2011), including the designation of allotropic forms[1].

1. Marriott, R. A. & Wan, H. H. Standard values of fugacity for sulfur which are self-consistent with the low-pressure phase diagram. *J. Chem. Thermodyn.* (2011). doi:10.1016/j.jct.2011.03.008
